# Termination of Pregnancy for Fetal Anomalies: A Retrospective Analysis of 112 Cases with Predictors of Late Termination

**DOI:** 10.3390/jcm15145699

**Published:** 2026-07-21

**Authors:** Süreyya Sarıdaş Demir, Serem Kel Ilgın, Mehmet Nuri Duran, Başak Nil Şen, İbrahim Eren Pek, Bülent Demir, Şenay Bengin Ertem, Fatma Sılan

**Affiliations:** 1 Department of Perinatology, Private Clinic, Çanakkale 17000, Türkiye; 2 Department of Obstetrics and Gynecology, Biga State Hospital, Çanakkale 17000, Türkiye; 3 Department of Obstetrics and Gynecology, Ezine State Hospital, Çanakkale 17000, Türkiye; 4 Department of Obstetrics and Gynecology, Faculty of Medicine, Çanakkale Onsekiz Mart University, Çanakkale 17000, Türkiye; 5 Department of Radiology, Faculty of Medicine, Çanakkale Onsekiz Mart University, Çanakkale 17000, Türkiye; 6 Department of Medical Genetics, Faculty of Medicine, Çanakkale Onsekiz Mart University, Çanakkale 17000, Türkiye

**Keywords:** termination of pregnancy, fetal anomaly, second trimester, anhidramnios, cardiovascular anomaly, late termination, predictors, Turkey

## Abstract

**Background and Objectives:** Termination of pregnancy (TOP) for fetal anomalies is a clinically and ethically complex intervention, and epidemiological data from regional Turkish referral centers are limited. This study aims to characterize the distribution of indications, identify predictors of late termination (gestational age ≥ 20 weeks), and analyze hospital resource utilization in a nine-year consecutive case series. **Materials and Methods:** A retrospective cohort study was conducted. All 112 cases of TOP for confirmed fetal anomalies or fetomaternal indications were included. Predictors of late termination were identified by binary logistic regression with three a priori selected variables (cardiovascular anomaly, CNS anomaly, maternal age; EPV [events-per-variable ratio] = 13.3). Hospital stay was compared between gestational age groups using the Mann–Whitney U test. **Results:** The mean gestational age at termination was 18.2 ± 4.0 weeks. Amniotic fluid disorders (predominantly anhidramnios, n = 35, 31.3%) were the single most frequent specific diagnosis. Cardiovascular anomaly was the only variable associated with late termination on logistic regression (OR 15.34, 95% CI 1.81–130.34; *p* = 0.012); CNS anomaly and maternal age were not significant. Cardiovascular anomalies were terminated at significantly later gestational ages than all other indication categories (pairwise Mann–Whitney U; Bonferroni-corrected *p* < 0.01 for four of five comparisons). Hospital stay was significantly longer in cases terminated at ≥20 weeks (median 3 vs. 2 days; *p* = 0.001). **Conclusions:** Amniotic fluid disorders dominate the indication profile at this center, distinguishing it from chromosomally focused Western series. Cardiovascular anomalies were associated with late termination, a finding consistent with the gestational age constraints of fetal echocardiography. These findings have implications for prenatal screening program design and healthcare resource planning.

## 1. Introduction

Termination of pregnancy (TOP) for fetal anomalies represents one of the most ethically sensitive and clinically demanding areas of perinatal medicine. Congenital anomalies are detected in approximately 2–3% of pregnancies, and a substantial proportion of couples opt for TOP following a confirmed prenatal diagnosis [1,2]. The indication spectrum, timing of termination, and procedural approach vary considerably across healthcare systems, reflecting differences in prenatal screening infrastructure, legal frameworks, and cultural context [3,4,5]. In Turkey, TOP is legally permitted beyond 10 weeks of gestation when a confirmed fetal anomaly or maternal health risk is documented, requiring formal approval by a multidisciplinary clinical committee [6,7]. As a result, most anomaly-related terminations in Turkey are performed in the second trimester.

The gestational age at which TOP is performed is critically determined by when the anomaly is detected. Central nervous system (CNS) anomalies are typically identifiable during the first trimester or early anatomy scan [8], whereas cardiovascular anomalies require dedicated fetal echocardiography performed at 20–22 weeks [9,10], inherently constraining the window for decision-making. This variation in detection timing—and its consequences for the gestational age at termination—has direct implications for counseling, procedural planning, and healthcare resource utilization [11].

Published series on TOP from Turkey have predominantly originated from major metropolitan centers and report chromosomal anomalies and CNS malformations as the leading indications. By contrast, data from non-metropolitan regional centers—which may serve populations with different screening uptake, socioeconomic characteristics, and referral patterns—are limited. Specifically, the contribution of amniotic fluid disorders (anhidramnios and oligohydramnios) to the overall TOP burden at regional centers has been incompletely characterized, representing a gap this study addresses.

This study reports a retrospective series of 112 consecutive TOP cases spanning nine years at tertiary referral centers in Çanakkale, northwestern Turkey—a non-metropolitan region with distinct referral and screening characteristics. Our primary aim is to identify independent predictors of late termination (gestational age ≥ 20 weeks) using binary logistic regression with a priori variable selection. Secondary aims include characterizing the distribution of specific diagnoses and analyzing predictors of hospital stay. This study was reported in accordance with the STROBE guidelines for observational studies [12]; a STROBE checklist is provided as Supplementary Material (Table S1).

## 2. Materials and Methods

### 2.1. Study Design and Setting

This study is a retrospective cohort study. All cases of termination for confirmed fetal anomalies or fetomaternal indications were identified through a systematic review of institutional medical records and operative logbooks.

### 2.2. Ethics

The study was approved by the Çanakkale Onsekiz Mart University Non-Invasive Clinical Research Ethics Committee (No: 2025-01/01-27; Meeting Date: 8 January 2025) and conducted in accordance with the principles of the Declaration of Helsinki. Individual patient consent was waived by the ethics committee due to the retrospective study design. No personally identifiable data are reported in this manuscript.

### 2.3. Inclusion and Exclusion Criteria

All women who underwent TOP for confirmed fetal anomalies or fetomaternal indications were included. Confirmation required ultrasonographic, chromosomal, or clinicopathological documentation. Cases terminated for purely socioeconomic or psychiatric indications without a confirmed anomaly, and cases with incomplete records precluding anomaly classification (n = 6) were excluded, yielding a final analytic cohort of 112 cases.

### 2.4. Variable Definitions

Indications were classified into six predefined categories: (1) CNS anomalies (including anencephaly, acrania, neural tube defects, hydrocephalus, and cerebellar malformations); (2) cardiovascular anomalies (including hypoplastic left heart syndrome, atrioventricular septal defects, truncus arteriosus, and pulmonary atresia); (3) genitourinary anomalies (renal agenesis or severe renal dysplasia presenting as anhidramnios); (4) syndromes/structural anomalies (including body stalk anomaly, Cantrell pentalogy, limb-body wall complex, VACTERL association, and Meckel–Gruber syndrome; trisomy 21 cases with coexisting major structural anomalies were classified here); (5) chromosomal/genetic conditions (including trisomy 21 without major structural co-anomaly, trisomy 18, trisomy 13, and monosomy X); and (6) maternal/other conditions, subdivided into amniotic fluid disorders (anhidramnios and severe oligohydramnios without an identifiable primary structural fetal anomaly) and other maternal indications (including HELLP syndrome, teratogenic medication exposure, and cervical incompetence). When a case presented with anomalies potentially spanning more than one category, the case was classified according to the clinically dominant indication, as determined by the treating perinatologist at the time of diagnosis. Late termination was defined as gestational age ≥ 20 weeks at the time of procedure. Termination procedures were classified as: (1) medical—misoprostol administration alone; (2) surgical—uterine evacuation by dilatation and curettage alone; or (3) combined medical–surgical—misoprostol-induced cervical ripening and labor/abortion induction followed by uterine evacuation (D&C), which is the predominant approach at this center [13]. The combined approach was employed irrespective of gestational age when complete uterine evacuation was required after medical induction.

### 2.5. Statistical Analysis

Continuous variables are reported as mean ± standard deviation (SD) or median (interquartile range, IQR) after assessment of distributional characteristics by the Shapiro–Wilk test. Categorical variables are reported as frequencies and percentages. Gestational age across indication categories was compared using pairwise Mann–Whitney U tests; given five pairwise comparisons involving the cardiovascular category, a Bonferroni-corrected significance threshold of α = 0.01 was applied to these comparisons. Hospital stay was compared between gestational age groups (<20 vs. ≥20 weeks) by Mann–Whitney U test (α = 0.05). All statistical analyses were performed using IBM SPSS Statistics, version 26.0 (IBM Corp., Armonk, NY, USA). Statistical significance was set at *p* < 0.05 unless otherwise stated.

Predictors of late termination were analyzed by binary logistic regression. Three variables were selected a priori based on clinical relevance and published literature: cardiovascular anomaly category, CNS anomaly category, and maternal age. The a priori selection was made to limit model complexity and avoid overfitting given the available sample size; additional variables including parity, fetal sex, and specific diagnostic subcategories were not included in the primary model to preserve parsimony. The events-per-variable (EPV) ratio—defined as the number of outcome events divided by the number of predictor variables—was 13.3, meeting the recommended minimum threshold of 10 for stable logistic regression estimates. Results are reported as odds ratios (OR) with 95% confidence intervals (CI).

AI-assisted language editing tools were used solely for English grammar and readability improvement of this manuscript.

## 3. Results

### 3.1. Demographics

A total of 112 cases were included. The mean maternal age was 30.8 ± 5.6 years (range 21–42). Mean gravidity was 2.0 ± 1.3 and mean parity was 0.7 ± 1.0. The mean gestational age at termination was 18.2 ± 4.0 weeks (range 10.4–26.0). Twenty-two cases (19.6%) were terminated before 14 weeks, 50 (44.6%) at 14–20 weeks, 34 (30.4%) at 20–24 weeks, and 6 (5.4%) at ≥24 weeks. All six cases terminated at or beyond 24 weeks involved confirmed lethal or surgically uncorrectable structural anomalies (cardiac defects not amenable to correction [n = 4] or bilateral renal agenesis [n = 2]) and received formal multidisciplinary committee approval in accordance with Turkish legislation (Kanun No. 2827) permitting TOP at any gestational age when a confirmed fetal anomaly or maternal health risk is documented.

### 3.2. Fetal Sex

Fetal sex was assessable in 88 of 112 cases (78.6%): 55 male (62.5%) and 33 female (37.5%). In 24 cases (21.4%), sex could not be determined. No statistically significant association between fetal sex and anomaly category was observed (Fisher’s exact test, all *p* > 0.05).

### 3.3. Indication Categories and Specific Diagnoses

The distribution of indication categories is presented in Table 1; specific diagnoses are detailed in Table 2. Amniotic fluid disorders—predominantly anhidramnios—constituted the single most frequent specific diagnosis across the cohort (n = 35, 31.3%), appearing within both the genitourinary category (n = 10, all secondary to suspected renal anomaly) and the maternal/other category (n = 25, without a primary structural anomaly). Anencephaly/acrania was the most frequent CNS diagnosis (n = 13 of 22 CNS cases). Among chromosomal and syndrome categories, trisomy 21 (n = 12 when including cases categorized as syndromes/structural, 10.9%) and trisomy 18 (n = 6, 5.5%) were most common.

### 3.4. Gestational Age by Indication Category and Late Termination

Gestational age at termination varied substantially across indication categories (Table 3). CNS anomalies were terminated at the earliest median gestational age (14.1 weeks, IQR 12.5–19.6). Cardiovascular anomalies were terminated at a significantly later gestational age than all other categories (median 23.0 weeks, IQR 22.0–24.0). After Bonferroni correction for five pairwise comparisons (adjusted α = 0.01), cardiovascular anomalies were terminated significantly later than CNS anomalies (*p* < 0.001), syndromes/structural anomalies (*p* = 0.008), chromosomal conditions (*p* = 0.001), and maternal/other conditions (*p* < 0.001); the comparison with genitourinary anomalies (*p* = 0.030) did not meet the Bonferroni-corrected threshold (Table 3). Cardiovascular anomalies also had the highest rate of late termination (8 of 9 cases, 88.9%).

### 3.5. Logistic Regression: Predictors of Late Termination

Of 112 cases, 40 (35.7%) met the definition of late termination (gestational age ≥ 20 weeks); all 112 cases had complete data for the three model covariates (indication category, maternal age). With an EPV of 13.3 (40 events/three predictors), the three-variable logistic regression model was appropriately powered (Table 4). Cardiovascular anomaly category was associated with late termination (OR 15.34, 95% CI 1.81–130.34; *p* = 0.012). CNS anomaly category (OR 0.58, 95% CI 0.19–1.74; *p* = 0.327) and maternal age per year (OR 0.99, 95% CI 0.91–1.06; *p* = 0.705) were not independently associated. The model’s Nagelkerke R^2^ was 0.152 and overall accuracy was 70.5%. Given the small cardiovascular subgroup (n = 9), the wide confidence interval should be interpreted with caution.

### 3.6. Procedure and Hospital Stay

The combined medical–surgical approach (misoprostol-induced labor/abortion followed by uterine evacuation) was employed in 87 of 109 cases with complete procedural data (79.8%) across all gestational age groups (Table 5); procedural records were unavailable for three cases due to transfer from an external center. Hospital stay was significantly longer in cases terminated at ≥20 weeks (median 3 days, IQR 2–4) than in those terminated before 20 weeks (median 2 days, IQR 2–3; *p* = 0.001). No significant difference in hospital stay was found between medical and combined procedural approaches (*p* = 0.542).

## 4. Discussion

This retrospective analysis of 112 consecutive TOP cases from tertiary referral centers in northwestern Turkey identifies cardiovascular anomaly as the strongest independent predictor of late termination and demonstrates that amniotic fluid disorders constitute the most frequent specific indication—a pattern distinct from the chromosomally dominated profiles reported in larger Western registry-based series.

The predominance of anhidramnios and severe oligohydramnios (n = 35, 31.8% of cases) is the most distinctive epidemiological finding of this cohort. This includes both genitourinary anhidramnios secondary to suspected renal anomaly (n = 10) and idiopathic anhidramnios without a primary structural anomaly (n = 25). Second-trimester anhidramnios carries a poor prognosis when associated with pulmonary hypoplasia or renal agenesis, and its high prevalence may reflect referral patterns at this center, local screening practices, and the late gestational age at which amniotic fluid abnormalities become apparent on routine ultrasound. The contribution of anhidramnios to the TOP burden in Turkish series has been previously noted, though it remains undercharacterized relative to the attention directed at chromosomal and CNS anomalies.

In the logistic regression model (three a priori selected variables; EPV = 13.3), cardiovascular anomaly was the only variable independently associated with late termination (OR 15.34, 95% CI 1.81–130.34; *p* = 0.012). This association is biologically plausible: complex structural cardiac defects require dedicated fetal echocardiography, typically performed at 20–22 weeks of gestation [14], thereby compressing the time available for diagnosis, counseling, and decision-making. In our cohort, 88.9% of cardiovascular anomaly cases were terminated at ≥20 weeks. Importantly, the wide confidence interval (1.81–130.34) reflects the small subgroup size (n = 9) and limits the precision of this estimate; the finding should be interpreted as directional evidence of association rather than a precise effect size. Future studies with larger cardiovascular anomaly cohorts are needed to confirm this association.

The significantly longer hospital stay associated with late termination (median 3 vs. 2 days; *p* = 0.001) has practical resource implications. Units receiving referrals for cardiovascular and other late-diagnosed anomalies should anticipate greater bed occupancy per case. The equivalence of hospital stay between medical and combined procedural approaches (*p* = 0.542) suggests that the additional surgical step does not independently prolong hospitalization.

This study has several limitations. The retrospective design and the absence of systematic postmortem pathological confirmation for all diagnoses introduces potential classification bias. The nine-year study period encompasses meaningful evolution in prenatal screening practices; temporal trend analysis was not performed, and secular changes in indication distribution or screening uptake cannot be excluded. The cardiovascular anomaly subgroup (n = 9) is small, limiting statistical precision. No data were available on prenatal counseling processes, parental decision-making, or post-procedure complications.

## 5. Conclusions

Amniotic fluid disorders represent the dominant specific indication at this regional center, a pattern distinct from Western series. Cardiovascular anomalies were associated with late termination—a finding consistent with the inherent gestational age constraints of fetal echocardiography—and with significantly longer hospital stay. These findings support consideration of earlier targeted cardiac screening and may inform resource planning at regional referral centers in Turkey. Larger prospective studies are needed to validate the predictors of late termination identified in this series.

## Figures and Tables

**Table 1:** Distribution of indication categories (n = 112).

Indication Category	n (%)	Late TOP ≥ 20w n (%)	Median GA (IQR), Weeks
CNS anomalies	22 (20.0%)	5 (22.7%)	14.1 (12.5–19.6)
Cardiovascular anomalies	9 (8.2%)	8 (88.9%) *	23.0 (22.0–24.0)
Genitourinary anomalies	10 (9.1%)	2 (20.0%)	18.4 (17.6–19.2)
Syndromes/Structural	24 (21.4%)	11 (40.7%)	18.2 (13.4–22.2)
Chromosomal/Genetic	13 (11.8%)	2 (15.4%)	18.1 (14.1–19.3)
Amniotic fluid disorders	24 (21.8%)	10 (41.7%)	19.2 (16.4–20.6)
Other maternal indications	4 (3.6%)	1 (25.0%)	15.1 (12.1–21.5)
**Total**	112 (100%)	39 (35.5%)	18.1 (14.6–20.9)

GA: gestational age; IQR: interquartile range; TOP: termination of pregnancy. * Significantly higher late TOP rate vs. all other categories (*p* < 0.05, pairwise comparisons).

**Table 2:** Specific diagnoses by indication category (n = 112).

Specific Diagnosis	n	%
**CNS anomalies (n = 22)**		
Anencephaly/acrania	13	11.8
Hydrocephalus/cerebellar hypoplasia	4	3.6
Neural tube defect (NTD)/spina bifida	4	3.6
Iniencephaly	1	0.9
**Cardiovascular anomalies (n = 9)**		
Hypoplastic left heart syndrome	2	1.8
Univentricular heart	2	1.8
Truncus arteriosus	2	1.8
AVSD/complex cardiac malformation	3	2.7
**Genitourinary anomalies (n = 10)**		
Anhidramnios secondary to renal anomaly	10	9.1
**Syndromes/Structural anomalies (n = 27)**		
Trisomy 21 with structural anomaly	6	5.5
Trisomy 18 with structural anomaly	3	2.7
Trisomy 13	2	1.8
Pentalogy of Cantrell	2	1.8
Body stalk anomaly	2	1.8
Other complex syndromes (VACTERL, Meckel-Gruber, etc.)	12	10.9
**Chromosomal/Genetic (n = 13)**		
Trisomy 21	6	5.5
Trisomy 18	4	3.6
Monosomy X with cystic hygroma/hydrops	1	0.9
Other (Trisomy 13, triploidy, Roberts syndrome)	2	1.8
**Amniotic fluid disorders (n = 24)**		
Idiopathic anhidramnios/severe oligohydramnios	22	20.0
Anhidramnios in twin gestation	2	1.8
**Other maternal indications (n = 4)**		
Teratogen drug exposure (category D)	2	1.8
Cervical insufficiency	1	0.9
HELLP syndrome	1	0.9
Total	112	100

AVSD: atrioventricular septal defect; VACTERL: vertebral, anal, cardiac, tracheoesophageal, renal, limb anomalies; HELLP: haemolysis, elevated liver enzymes, low platelets. “Trisomy 21 with structural anomaly” cases were classified under Syndromes/Structural due to co-existing major malformations.

**Table 3:** Gestational age at termination by indication category with pairwise comparisons.

Category	n	Mean GA (Weeks ± SD)	Median GA (IQR)	*p* vs. CVS
CNS anomalies	22	16.1 ± 4.0	14.1 (12.5–19.6)	<0.001
Cardiovascular	9	22.6 ± 2.2	23.0 (22.0–24.0)	—
Genitourinary	10	19.1 ± 2.4	18.4 (17.6–19.2)	0.030
Syndromes/Structural	27	17.5 ± 4.4	18.2 (13.4–22.2)	0.008
Chromosomal/Genetic	13	17.2 ± 3.1	18.1 (14.1–19.3)	0.001
Amniotic fluid disorders	24	19.5 ± 2.8	19.2 (16.4–20.6)	<0.001
Other maternal	4	15.7 ± 3.8	15.1 (12.1–21.5)	<0.001
**Overall**	112	18.2 ± 4.0	18.1 (14.6–20.9)	

GA: gestational age; SD: standard deviation; IQR: interquartile range; CVS: cardiovascular. *p*-values from Mann–Whitney U test (pairwise vs. cardiovascular category).

**Table 4:** Binary logistic regression—predictors of late termination (GA ≥ 20 weeks; n = 112, events = 40, EPV = 13.3).

Variable	β	SE	OR (95% CI)	*p*-Value
Maternal age [standardized]	−0.128	0.218	0.99 (0.91–1.06)	0.705
CNS anomaly (ref: maternal/other)	−0.564	0.567	0.58 (0.19–1.74)	0.327
Cardiovascular anomaly (ref: maternal/other) *	2.712	1.092	15.34 (1.81–130.34)	0.012

OR: odds ratio; CI: confidence interval; SE: standard error; EPV: events per variable; ref: reference category. Nagelkerke R^2^ = 0.152; overall accuracy = 70.5%. * *p* < 0.05. The wide CI for cardiovascular anomaly reflects the small subgroup size (n = 9) and should be interpreted accordingly.

**Table 5:** Termination procedures by gestational age group (n = 112).

GA Group	Total n (%)	Medical n (%)	Surgical n (%)	Medical + Surgical n (%)
<14 weeks	22 (20.0%)	3 (13.6%)	1 (4.5%)	18 (81.8%)
14–20 weeks	48 (43.6%)	11 (22.9%)	0 (0.0%)	37 (77.1%)
20–24 weeks	34 (30.9%)	6 (17.6%)	0 (0.0%)	28 (82.4%)
≥24 weeks	5 (4.5%)	1 (20.0%)	0 (0.0%)	4 (80.0%)
**Total**	112 (100%)	21 (19.1%)	1 (0.9%)	87 (79.8%)

GA: gestational age. Hospital stay ≥ 20 weeks vs. <20 weeks: median 3 vs. 2 days (*p* = 0.001, Mann–Whitney U). No significant difference between medical and combined approaches (*p* = 0.542).

## Data Availability

The anonymized dataset is available from the corresponding author upon reasonable request.

## Supplementary Materials

The following supporting information can be downloaded at: https://www.mdpi.com/article/10.3390/jcm15145699/s1, Table S1: STROBE Checklist for Cohort, Case-Control, and Cross-Sectional Studies.
