# Supplementary material for: Termination of Pregnancy for Fetal Anomalies: A Retrospective Analysis of 112 Cases with Predictors of Late Termination
Source: J Clin Med. 2026 Jul 21;15(14):5699. doi: 10.3390/jcm15145699 (PMC13412660; doi:10.3390/jcm15145699)
Supplement: Supplementary file 1 [file jcm-15-05699-s001.zip › jcm-4387160-supplementary.pdf]

Table S1. STROBE Checklist for Cohort, Case-Control, and Cross-Sectional Studies

(Applicable checklist items for cross-sectional/retrospective cohort study design)

Section/Topic	Item No.	Recommendation	Reported on page/line No.
Title and abstract	1a	Indicate the study's design with a commonly used term in the title or the abstract	Title; Abstract
	1b	Provide in the abstract an informative and balanced summary of what was done and what was found	Abstract
**Introduction**			
Background/rationale	2	Explain the scientific background and rationale for the investigation being reported	Introduction, paragraphs 1–3
Objectives	3	State specific objectives, including any prespecified hypotheses	Introduction, final paragraph
**Methods**			
Study design	4	Present key elements of study design early in the paper	Methods, Study design
Setting	5	Describe the setting, locations, and relevant dates, including periods of recruitment, exposure, follow-up, and data collection	Methods, Study design; Table 1
Participants	6a	Give the eligibility criteria, and the sources and methods of selection of participants	Methods, Study design; Figure (STROBE flow diagram)
	6b	For matched studies, give matching criteria and number of exposed and unexposed (N/A for this study)	N/A
Variables	7	Clearly define all outcomes, exposures, predictors, potential confounders, and effect modifiers. Give diagnostic criteria, if applicable	Methods, Variables and definitions
Data sources/measurement	8*	For each variable of interest, give sources of data and details of methods of assessment. Describe comparability of	Methods, Variables and definitions

		assessment methods if there is more than one group	
Bias	9	Describe any efforts to address potential sources of bias	Methods; Discussion, Limitations
Study size	10	Explain how the study size was arrived at	Methods, Statistical analysis (EPV = 13.3)
Quantitative variables	11	Explain how quantitative variables were handled in the analyses. If applicable, describe which groupings were chosen and why	Methods, Statistical analysis
Statistical methods	12a	Describe all statistical methods, including those used to control for confounding	Methods, Statistical analysis
	12b	Describe any methods used to examine subgroups and interactions	Methods, Statistical analysis (Bonferroni correction)
	12c	Explain how missing data were addressed	Methods, Statistical analysis; Results 3.1
	12d	If applicable, describe analytical methods taking account of sampling strategy (cross-sectional/cohort specific)	Methods, Statistical analysis
	12e	Describe any sensitivity analyses	N/A (small subgroup noted; CI interpreted with caution)
**Results**			
Participants	13a	Report numbers of individuals at each stage of study (e.g., numbers potentially eligible, examined for eligibility, confirmed eligible, included in the study, completing follow-up, and analysed)	Results 3.1; STROBE flow diagram
	13b	Give reasons for non-participation at each stage	Results 3.1; Methods
	13c	Consider use of a flow diagram	Figure S1 (STROBE flow diagram) — recommended for revision
Descriptive data	14a	Give characteristics of study participants (e.g., demographic, clinical, social) and information on exposures and potential confounders	Results 3.1; Table 1

	14b	Indicate number of participants with missing data for each variable of interest	Results 3.1; Table 1
	14c	Summarise follow-up time (cohort specific — N/A)	N/A
Outcome data	15*	Report numbers of outcome events or summary measures over time (cohort specific)	Results 3.2–3.6; Tables 2–5
Main results	16a	Give unadjusted estimates and, if applicable, confounder-adjusted estimates and their precision (e.g., 95% CI). Make clear which confounders were adjusted for and why they were included	Results 3.5; Table 4
	16b	Report category boundaries when continuous variables were categorized	Methods; Table 3 (GA ≥20 weeks)
	16c	If relevant, consider translating estimates of relative risk into absolute risk for a meaningful time period	N/A
Other analyses	17	Report other analyses done — e.g., analyses of subgroups and interactions, and sensitivity analyses	Results 3.3 (Bonferroni pairwise); Results 3.5
**Discussion**			
Key results	18	Summarise key results with reference to study objectives	Discussion, paragraphs 1–2
Limitations	19	Discuss limitations of the study, taking into account sources of potential bias or imprecision. Discuss both direction and magnitude of any potential bias	Discussion, Limitations paragraph
Interpretation	20	Give a cautious overall interpretation of results considering objectives, limitations, multiplicity of analyses, results from similar studies, and other relevant evidence	Discussion; Conclusion
Generalisability	21	Discuss the generalisability (external validity) of the study results	Discussion, Limitations paragraph
**Other information**			
Funding	22	Give the source of funding and the role of the funders for the	Funding statement

	present study and, if applicable, for the original study on which the present article is based	

** Items marked with an asterisk are modified for cross-sectional study design. N/A = not applicable. GA = gestational age. CI = confidence interval. STROBE = Strengthening the Reporting of Observational Studies in Epidemiology.*
